# Supplementary material for: A Comparative Analysis of the Polyphenolic Content and Identification of New Compounds from Oenothera biennis L. Species from the Wild Flora
Source: Molecules. 2025 Oct 11;30(20):4059. doi: 10.3390/molecules30204059 (PMC12566031; doi:10.3390/molecules30204059)
Supplement: Supplementary file 1 [file molecules-30-04059-s001.zip › molecules-3877113-supplementary.pdf]

Supplementary Materials

A Comparative Analysis of the Polyphenolic Content and Identification of New Compounds from *Oenothera biennis* L. Species from the Wild Flora

Viviane Beatrice Bota [1,2,3,*,†], Neli-Kinga Oláh [4,5], Elisabeta Chișe [5], Ramona-Flavia Burtescu [4], Flavia-Roxana Pripon Furtună [4], Lăcrămioara-Carmen Ivănescu [1], Maria-Magdalena Zamfirache [1], Endre Máthé [2,6] and Violeta Turcuș [2,3,*,†]

1 Doctoral School of Biology, Faculty of Biology, "Alexandru Ioan Cuza" University of Iași, 700505 Iasi, Romania; ivanescu@uaic.ro (L.-C.I.); magda@uaic.ro (M.-M.Z.)
2 Faculty of Medicine, "Vasile Goldiș" Western University of Arad, 310414 Arad, Romania; endre.mathe@agr.unideb.hu
3 National Institute for Economic Research "Costin C. Kirițescu", Romanian Academy, Centre for Mountain Economy (CE-MONT), 725700 Suceava, Romania
4 SC PlantExtrakt SRL, Rădaia, 407059 Cluj, Romania; neliolah@yahoo.com (N.-K.O.); ramona.burtescu@plantextrakt.ro (R.-F.B.); flavia.pripon@plantextrakt.ro (F.-R.P.F.)
5 Faculty of Pharmacy, "Vasile Goldiș" Western University of Arad, 310414 Arad, Romania; chise.elisabeta@uvvg.ro
6 Institute of Nutrition, Faculty of Agricultural and Food Sciences and Environmental Management, University of Debrecen, H-4032 Debrecen, Hungary
* Correspondence: viviane.beatrice@gmail.com (V.B.B.); turcus.violeta@uvvg.ro (V.T.)
† These authors contributed equally to this work and are both considered the first author.

Table S1. Phytochemical composition of *O. biennis* L. - summarized results of original articles sorted by chronological appearance, plant part, and extraction method.

Plant part	Type of extract/analyzed material	Compounds	References
**Not specified**	Not specified	Kaempferol 3-O-glu, 3-O-rha glu, quercetin 3-O-glu, 3-O-gal, 3-O-ara și 3-O-rha glu	[10]
**[13]Leaves**	Hydrolyzed acetone extract	Ellagic acid, gallic acid, digallic acid, neochlorogenic acid, caffeic acid, *p*-coumaric acid, *o*-coumaric acid, quercetin, kaempferol, delphinidin, cyanidin	[11]
	Hydroalcoholic extract	Oenothein A	[12]
	Hydroacetone extract	Elagitanins, Oenothein A and B, and isomers of oenothein A, caffeoyl tartaric acid, quercetin glucuronide, flavonoid glycoside, kaempferol glucuronide	[13]
	Hexane chloroform-methanol extract	Cholesterol, brassicasterol, campsterol, stigmasterol, beta-sitosterol	[14]
	Methanolic extract	Carbohydrates, glycosides, alkaloids, sterols, triterpenoids, tannins, flavonoids, fixed oils	[15]
	Hydroacetone extract and ellagitannin fractions	Ellaginatins–Oenothein A and B, oligomers of Oenothein	[16,17,18]
	Volatiles from solid-phase microextraction	Aminoacids<br>Infested with *Altica* larvae: Isisovaleronitrile, (E)- and (Z)-isovaleraldoxime, 3-methyl-1-nitrobutane, alpha-pinene,	[19]

			(E)-beta-ocimene, 3-methylbutyl isovalerate, Germacrene D, 9E,E)-alpha-farnesene	
		Infested with *Altica*	Without 3-methylbutyl isolavalerate and Germacrene D, + DMNT	
		Treated with MeJA	All compounds mentioned in the study	
	Raw polysaccharide extract		Heteropolysaccharides–arabinose, mannose, galactose, talose	[20]
	Hydromethanolic extract		Methyl ester 10-octadecenoic acid, 4H-1-bezopyran-4-one-7-hydroxy-2-(4-hydroxyprenyl), caryophyllene, oleic acid, 2,6-bis(1,1-dimethyl)-4-[(4-hydroxy-3,5-dimethylphenyl)methyl]-phenol, 4-methyl-1-(1-methylethyl)-3-cyclohexen-1-ol, 3-Buten-2-one, 4-(2,5,6,6-tetramethyl-2-cyclohexan-1-yl), a-ketostearic acid, 4,8,12,16-Tetramethylheptadecan-4-olide, isopropyl stearate, chromaryl	[21]
**Leaves**	Lipid fractions		Alpha-linolenic acid, linoleic acid, palmitic acid	[22]
**Seeds**			Fatty acids–gamma-linolenic, alpha-linolenic, oleic acid, phospholipids, glycolipids, triacylglycerols, acyl fractions	
**Herba**	Free phenolic acid fractions, fractions after acid and alkaline hydrolysis		Salicylic, p-hydroxybenzoic, protocatechuic, vanillic, gentusic, p-coumaric, caffeic, ferulic, p-hydroxyphenylacetic, syringic, gallic, pyrocatechuic, 2-hydroxy-4-methoxybenzoic acids	[23]
	Phenolic acid fractions (free, hydrolyzed, alkaline hydrolyzed)		Gallic, caffeic, protocatechuic, gentisic (only hydrolysed), gamma-resorcylic and p-hydroxyphenylacetic (hydrolysed), p-hydroxybenzoic, p-coumaric, pyrocatechuic (free), vanillic, syringic, ferulic, salicylic (free) acids	[24]
	Aqueous extract with acetonitrile		Elagitanins–Oenothein B	[25]
	Raw extract obtained from filtered and freeze-dried methanolic extract		Oenothein B; glycosides and glucuronides of quercetin and kaempferol; myricetin glucuronide; phenolic acids; 3- and 4-caffeoylquinic acid, 3-p-feruloylquinic acid, 4-p-coumaroylquinic acid, 4-p-feruloylquinic acid	[26]
	Sonicated and filtered ethanolic extract		Gallic acid, caffeic acid, epicatechin, coumaric acid, ferulic acid, rutoside, rosmarinic acid	[27]
**Herba and root**	Essential oil (neutral fractions, soluble sodium bicarbonate, soluble sodium hydroxide)		79 compounds, furfural (major constituent), alpha-pinene, camphene, beta-pinene, beta-myrcene, limonene, isoamyl alcohol, n-amyl alcohol, n-hexanol, cis-3-hexen-1-ol, n-nonyl, aldehydes, 1-octen-3-ol, n-heptanol, trans-linalool oxide, cis-linalool oxide, benzaldehyde, furfuryl acetate, linalool, n-octanol, linalyl acetate, furfuryl alcohol, beta-caryophyllene, alpha-terpineol, benzyl acetate, alpha-terpinyl acetate, gamma-muurolene, benzyl alcohol, omega-cardinene, gamma-caryophyllene, calamenene, beta-phenyl ethyl alcohol, n-C10~C38 aliphatic hydrocarbons, n-C4~C18 aliphatic fatty acids, 2-furan carboxylic acid, benzoic acid, oleic acid, linoleic acid, phenol,	[28]

		o-cresol, p-cresol, eugenol, thymol	
Root	n-Hexane and ethyl acetate fractions of the methanolic extract	Oleanolic acid, maslinic acid, sitosterol, galic acid, 2,7,8-trimethylellagic acid, tetramethylellagic acid, 2-methyl-7-oxo-tritetracont-1, 5-dien-2l-ol, a18-hydroxypentacos-2l-en-I-oic acid, 5-methyl-27-oxo-tricont-4-en-24-ol, acid 3,5-dihydroxy-4-pent-4′-enoil-1′-oxymethybenzoic acid (oenostacin)	[29]
	Ethanolic extract	Oenostacin	[30]
	Methanolic extract	Dihydroxyprenylxanthone, cetoleyl diglucoside, oenotheralanosterol A and B, oenotherafenoxylactone, oenotheafitilactone	[31]
	Methanolic extract	Oenoteralansterol A and B, cetoleyl diglucoside, oenotheraphenoxylactone, acetylated dihydroxyprenylxanthone, dihydroxyprenylxanthone, oenotheraphitylactone	[32]
	Methanolic extract	Benzoic acid, diterpene acid, tetralin lactone, dodecenyl benzene triol, prenyl anthracene diol, acyl diglucoside	[33]
Whole plant	Aqueous extract	Alkaloids, flavonoids, saponins, tannins	[34]
	Ethanolic extract	Alkaloids, oils, flavonoids, saponins, glycosides	
	Ethyl acetate extract	Alkaloids, oils, flavonoids, glycosides, tannins	
Seeds	Dried seeds, oil	Seeds–water, protein, oil, fiber, starch, dextrins, and sugars; Amino acids: tryptophan, lysine, threonine, cysteine/methionine, valine, isoleucine, leucine, tyrosine/phenylalanine; Ca, Mg, K, P, Mn; linolenic acid, gamma-linolenic acid; sterols (cholesterol, capesterol, beta-sitosterol), 4-methylsterols (obtrusifoliol, gramisterol, citrostadienol), triterpene alcohols (beta and alpha amyrin, c-artenol, methylene-c-artanol), tocopherols (alpha, gamma, omega)	[35]
	Oil–triglycerides isolated with benzene-methanol	Fatty acids - myristic, palmitic, palmitoleic, stearic, oleic, linoleic, gamma-linolenic - LLL, LLO, LLP, LOO, LOP, LLS, gammaLnLp, LOS, gammaLnLS, gammaLnLL, LPP, OOO, LPS, gammaLnLO; tristearin, glycerol 1,3-stearate 2-oleate, triolein, glycerol 1-palmitate 2-oleate 3-linoleate, triolein, trilinolenin	[36]
	Oil, esters, and methyl esters separated	Saturated fatty acids (C12-C24), monounsaturated fatty acids (C18:1 (n-9) major constituent), polyunsaturated fatty acids (C18:3 (n-6) C18:2 (n-6) major constituents)	[37]
	Free phenolic acid fractions, fractions after acid and alkaline hydrolysis	Acids: ferulic, vanilic, siringic, p-coumaric, p-hidroxifenilacetic, cafeic, protocatechuic, galic, pirocatechuic, salicilic, 2-hidroxi-4-metoxibenzoic	[23]
	Phenolic acid fractions (free, hydrolyzed, alkaline hydrolyzed)	Acids: gallic, caffeic, protocatechuic, p-hydroxyphenylacetic (only hydrolyzed), p-hydroxybenzoic, p-coumaric, pyrocatechuic and o-coumaric (free), vanillic, syringic (hydrolysate), ferulic, salicylic, 2-hydroxy-4-methoxybenzoic	[24]
	Oil	Gamma-linolenic acid, unsaponifiable matter, dimethylsterols, triterpene alcohols (lupeol, 7,24-tirucaladienol, cycloartenol, butirospermol, 24-methylene cycloartanol, germanicol	[38]

**Defatted seeds**	Phenolic acid fraction	Acids: p-Hydroxyphenylacetic, p-hydroxybenzoic, 2-hydroxy-4-methoxybenzoic, caffeic, hydroxycaffeic, m-coumaric, p-coumaric, ferulic, gallic, protocatechuic, vanillic, veratric, homoveratric, salicylic	[39]
	Ethanolic extract	Penta-O-galloyl-beta-D-glucose, gallic acid, catechin, procyanidin B1, procyanidin B3, proanthocyanidins	[40]
**Seeds**	Oil (unrefined, cold pressed)	Lipophilic triterpenoid esters, 3-O-trans-caffeoyl derivatives of betulinic, morolic, and oleanolic acids	[41]
	Oil	Fatty acids–linoleic and gamma-linolenic; phytosterols–beta-sitosterol; tocopherols (alpha and gamma)	[42]
	Saponifiable fractions, unsaponifiable fractions, methanolic extract	Tocopherols; phytosterols: brassicasterol, campesterol, stigmasterol, sitosterol, avenasterol; phenols (736.47 +/- 0.98 mg/100 g)	[43]
	Unsaponifiable matter from oil	Sterols–alpha-colestanol, campsterol, beta-sitosterol, sitostanol, delta5-avenasterol, delta7-avenasterol	[44]
	Oil	Fatty acids: oleic, linoleic, gamma-linolenic + palmitic, stearic, vaccenic, eicosanoic, docosanoic;<br>Phytosterols: beta-sitosterol, campesterol, delta5-avenasterol, D5-24-stigmastadienol, D7-stigmasterol, D7-avenasterol; erythrodiol, uvaol;<br>Linear aliphatic alcohols (C21-C-28, geraniol, phytol), triterpene alcohols (dammaradienol, taraxterol, beta-amyrin, butirospermol, cycloartenol, 24-methylcycloartenol; citrostadienol; squalene, lauryl arachidate, methyl heptadecanoate; Aliphatic and terpenic waxes;<br>Phenols: ferulic acid, vanillin, hydroxytyrosol and derivatives, vanillic acid, p-coumaric acid	[45]
	Oil—long-chain fatty acid fractions	Eicosanol, docosanol, tricosanol, tetracosanol, pentacosanol, hexacosanol, heptacosanol, octacosanol	[46]
**Seeds and sprouts, respectively**	Lyophilized form digested with sulfuric acid and hydrogen peroxide; methanolic extract	Minerals (Na, K, Ca, Mg, Fe, Mn, Cu, Zn); gallic acid (in sprouts), caffeic acid, p-coumaric acid, ferulic acid	[47]
**Seeds**	Oil	Fatty acids–palmitic, stearic, oleic, linoleic, linolenic, eicosenoic acids; sterols, alcohols, hydrocarbons, squalene, vitamin E, phytol, farnesol, amyrin, campesterol, stigmasterol, beta-sitosterol, lanosterol, alpha, gamma, delta tocopherols	[48]
	Oil	Unsaturated fatty acids–oleic acid, linoleic acid, gamma-linolenic acid; Saturated fatty acids: palmitic acid, stearic acid; proteins	[49,50]
	Oil	Palmitic acid, stearic acid, alpha-linolenic acid, linolenic acid, gamma linolenic acid, oleic acid, phytosterols (4-desmethylsterol, erythrodiol, uvaol), phenols (ferulic acid), tocopherols	[51]
	Degreased with n-hexane	Syringic anhydride, trimethylenglykol-digalloat, monogalloylglucose,	[52]

	and integral	ellagic acid glycoside, ellagic acid xyloside, syringic acid, ellagic acid, quercetin glucuronide, and kaempferol glucuronide, protocatechuic acid, procyanidin B, procyanidin trimer, (+)-catechin, methyl gallate, procyanidin B gallate, catechin gallate, galloylxy trihydroxyflavanone, quercetin xylopyranoside, methyl ellagic acid, quercetin; Sugars, amino acids, minerals, organic acids (oxalic, citric), and tocopherols (alpha, beta, gamma)	
	Commercial oil	8 markers	[53]
**Seed meal**	Ethanol-ethyl acetate extract, ethanolic extract, hydroacetone extract	Methyl and ethyl derivatives of protocatechuic acid, gallic acid, catechin; epicatechin, procyanidin gallate, tannins	[54]
	Raw ethanol extract, ethyl acetate, and fractions	Gallic acid, catechin, syringic acid, ferulic acid, vanolic acid, caffeic acid (+ 9 unidentified compounds)	[55]
**Seed cake**	Ethanolic extract, with and without hydrolysis	Total phenols (466.67 mg GAE/g), Flavonoids (211.11 mg QE/g), reducing sugars (277.78 mg glucose/g)	[56]
	Acetone extract	Phenols, vanillin, catechin, epicatechin, gallic acid	[57]
**Deffated seed cake**	Ultrasound-assisted alkaline extraction	Edible proteins rich in essential amino acids (His, Ile, Leu, Lys, Met, Cys, Phe, Tyr, Thr, Trp, Val) and non-essential amino acids (Ala, Asp, Arg, Gly, Glu, Pro, Ser)	[58]
**Sprouts**	Hydroethanolic extracts (0%, 30%, 50%, 70%, 90%)	Ellagic acid (101–407 µg/mL), quercetin-3-O-glucuronide (96–381 µg/mL)	[59]
	50% hydroethanolic extract	Gallic acid, ellagic acid, miquelianin, luteolin-7-glucuronide	[60]

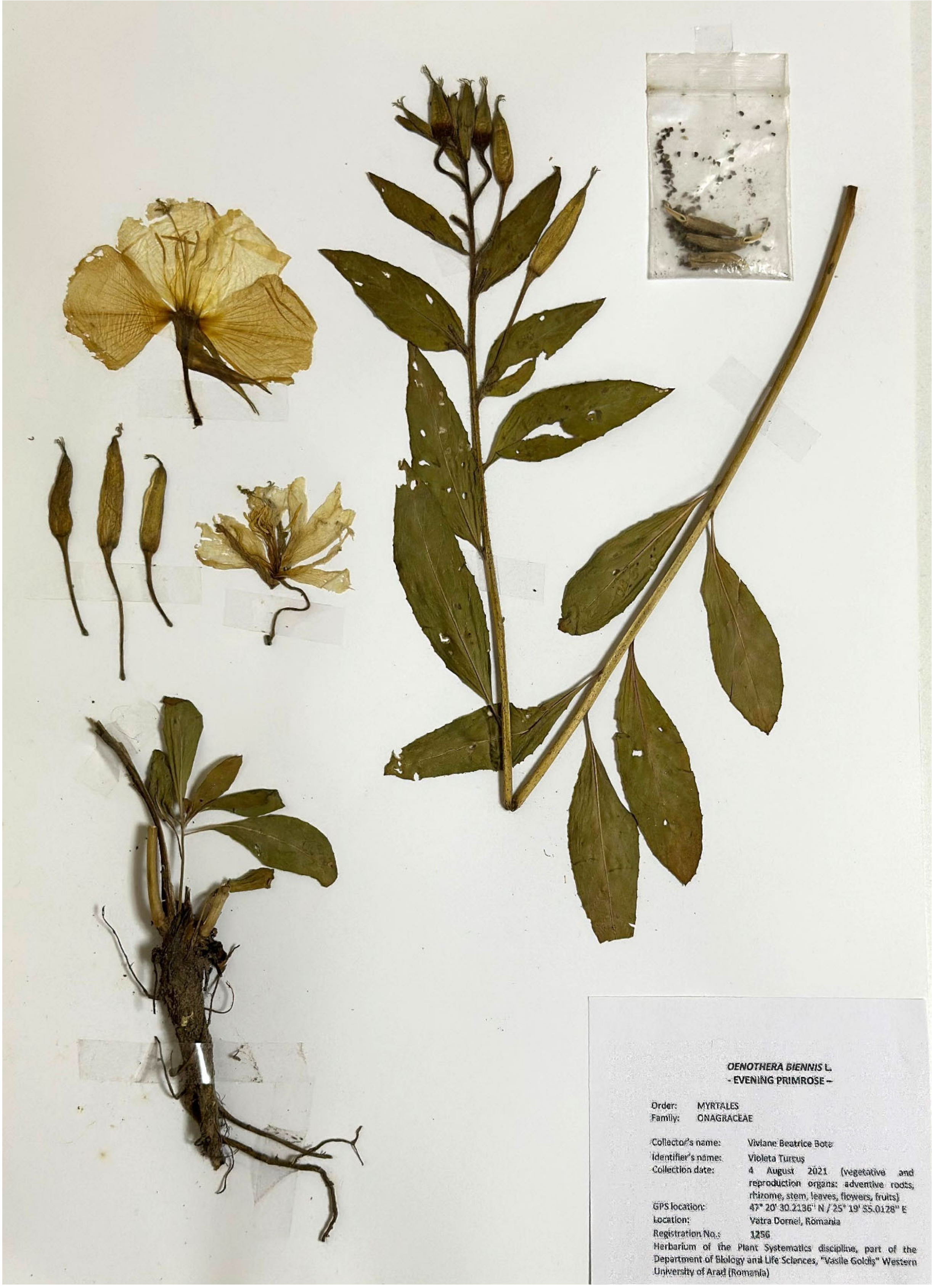

Figure S1. *Oenothera biennis* L. collected from Vatra Dornei. Registered with the voucher number 1256 in the Herbarium of the Plant Systematics discipline, part of the Department of Biology and Life Sciences of the "Vasile Goldiș" Western University of Arad (Romania).

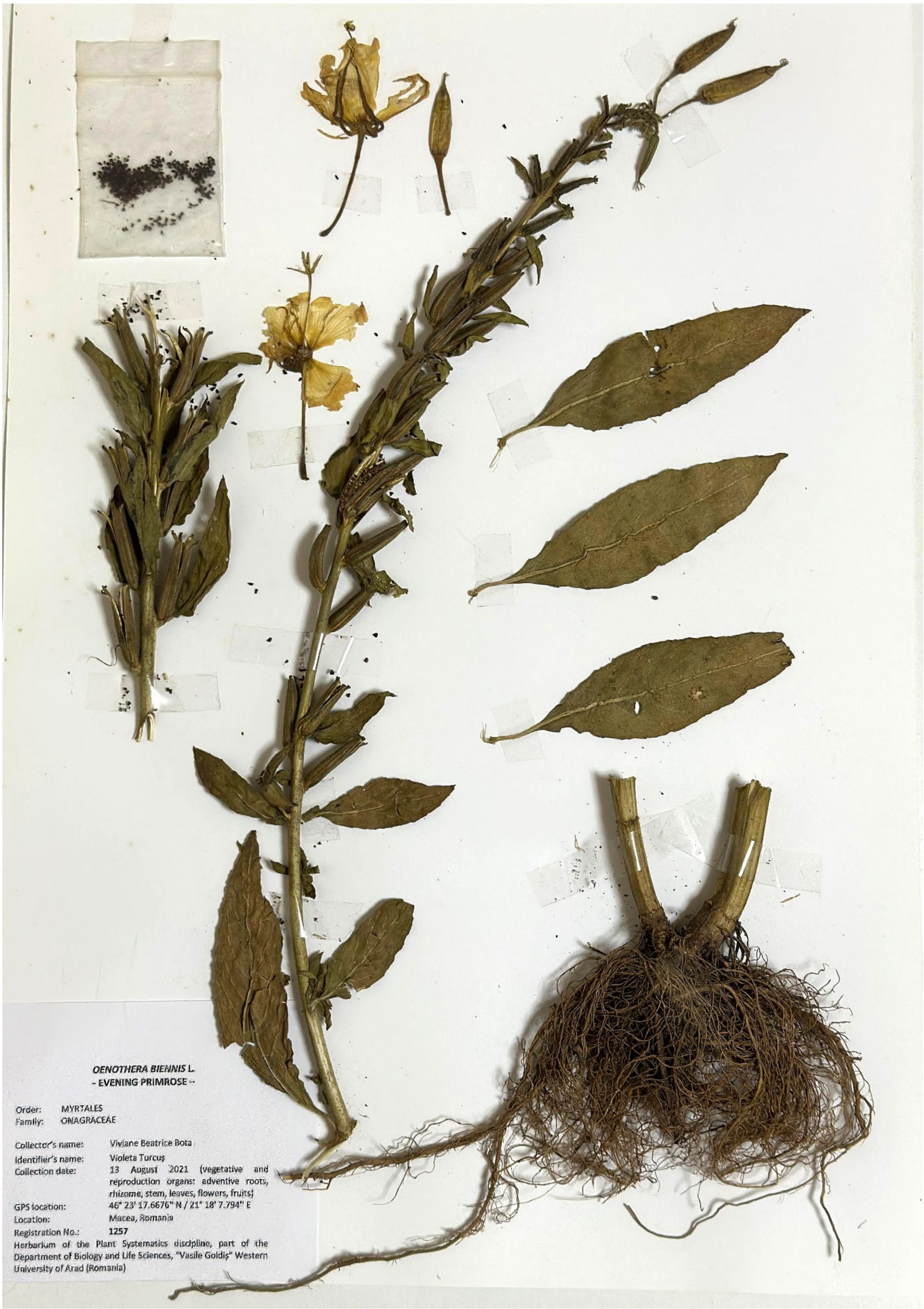

Figure S2. *Oenothera biennis* L. collected from Macea. Registered with the voucher number 1257 in the Herbarium of the Plant Systematics discipline, part of the Department of Biology and Life Sciences of the "Vasile Goldiș" Western University of Arad (Romania).

Table S2. Prior reports in *Oenothera biennis* L. species, by plant part and extract type, of compounds identified in the present study.

Compound	Compound derivative	Plant part	Type of extract	References
Caffeic acid		Leaves	Hydrolyzed acetone extract	[11]
		Herba	Sonicated and filtered ethanolic extract	[27]
		Seeds and sprouts, respectively	Lyophilized form digested with sulfuric acid and hydrogen peroxide; methanolic extract	[47]
		Seed meal	Raw ethanol extract, ethyl acetate, and fractions	[55]
Chlorogenic acid		Seeds	-	[95]
-	Neochlorogenic acid	Leaves	Hydrolyzed acetone extract	[11]
Salicylic acid		Herba	Free phenolic acid fractions, fractions after acid and alkaline hydrolysis	[23]
		Herba	Phenolic acid fractions (free, hydrolyzed, alkaline hydrolyzed)	[24]
		Seeds		
		Defatted seeds	Phenolic acid fraction	[39]
Gallic acid		Leaves	Hydrolyzed acetone extract	[11]
		Herba	Free phenolic acid fractions, fractions after acid and alkaline hydrolysis	[23]
		Herba	Phenolic acid fractions (free, hydrolyzed, alkaline hydrolyzed)	[24]
		Seeds		
		Herba	Sonicated and filtered ethanolic extract	[27]
		Defatted seeds	Ethanolic extract	[40]
		Seeds	Degreased with n-hexane and integral	[52]
		Seed meal	Ethanol-ethyl acetate extract, ethanolic extract, hydroacetone extract	[54]
		Seed meal	Raw ethanol extract, ethyl acetate, and fractions	[55]
		Seed cake	Acetone extract	[57]
		Sprouts	Hydroethanolic extract 50%	[60]
		Seeds and sprouts, respectively	Lyophilized form digested with sulfuric acid and hydrogen peroxide; methanolic extract	[47]
Apigenin		-	-	Prior report not found
Chrysin		-	-	Prior report not found
Esculetin		-	-	Prior report not found
Hesperetin		-	-	Prior report not found
Hyperoside		-	-	Prior report not found

Kaempferol		Leaves	Hydrolyzed acetone extract	[11]
		Leaves	Hydroacetone extract	[13]
		Herba	Raw extract obtained from filtered and freeze-dried methanolic extract	[26]
		Seeds	Degreased with n-hexane and integral	[52]
Luteolin	-	-	-	Prior report not found
-	Luteolin-7-glucuronide	Sprout	Dried ethanolic extract	[96]
Naringenin		-	-	Prior report not found
Quercetin		Leaves	Hydrolyzed acetone extract	[11]
		Leaves	Hydroacetone extract	[13]
		Herba	Raw extract obtained from filtered and freeze-dried methanolic extract	[26]
		Seeds	Degreased with n-hexane and integral	[52]
Rutoside		Herba	Sonicated and filtered ethanolic extract	[27]

Table S3. Mobile phase composition for LC/MS analysis.

Time, min	Methanol	Water	2 % formic acid in water
0.00	5	90	5
3.00	15	70	15
6.00	15	70	15
9.00	21	58	21
13.00	21	58	21
18.00	30	41	29
22.00	30	41	29
26.00	50	0	50
29.00	50	0	50
29.01	5	90	5
35.00	5	90	5

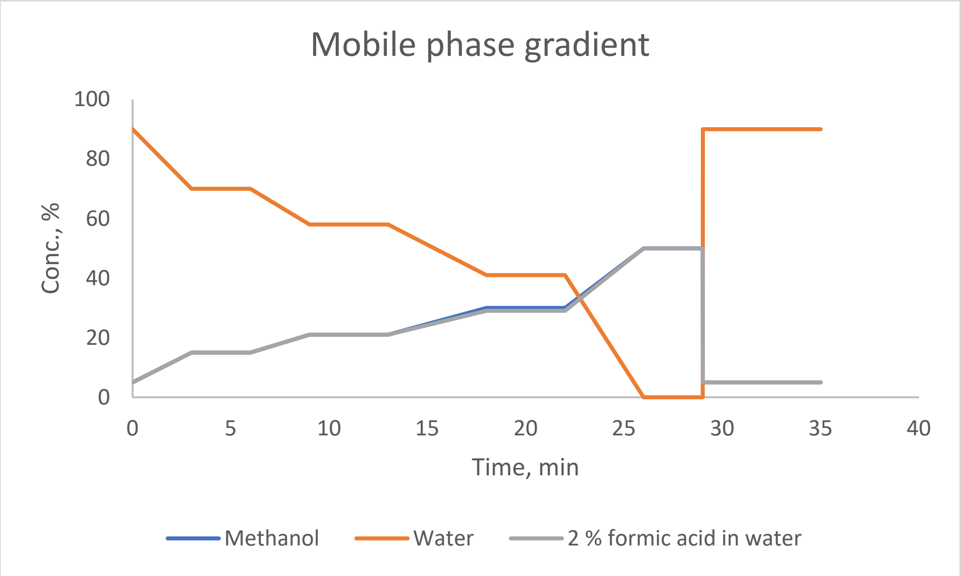

Figure S3. Graphical representation of the mobile phase gradient of LC/MS analysis.

Table S4. The standards for chromatographic and spectral data.

Name of standard	Retention time, min	m/z, and main transition	MRM	Other transitions
Caffeic acid	13.8	179.0>135.0	Negative	179.0>134.0<br>179.0>89.0
Chlorogenic acid	11.9	353.0>191.0	Negative	
Salicylic acid	23.5	137.0>93.0	Negative	137.0>75.0<br>137.0>65.0
Gallic acid	7.0	168.9>125.0	Negative	
Chrysin	29.7	253.0>143.0	Negative	253.0>119.0<br>253.0>107.0
Hyperoside	20.3	463.1>300.0	Negative	463.1>301.0
Kaempferol	27.9	285.0>187.0	Negative	285.0>151.0<br>285.0>133.0
Rutoside	20.2	609.0>300.0	Negative	609.0>301.0<br>609.0>271.0
Esculetin	13.0	179.1>123.0	Negative	
Hesperetin	27.0	301.0>164.0	Negative	301.0>136.0<br>301.0>108.0
Luteolin	26.8	287.0>153.0	Positive	
Naringenin	26.2	271.0>119.0	Negative	271.0>107.0
Quercetin	25.4	300.9>151.0	Negative	300.9>121.0
Apigenin	28.1	269.0>117.0	Negative	

Table S5. The standards used for LC/MS analyses.

Name of standard	Origin	Concentration range, mg/ml	Calibration curve equation	Correlation factor	Detection limit, mg/ml	Quantification limit, mg/ml
Caffeic acid	Phytolab, Vestenbergsgreuth, Germany	0.11–1.10	Area = $4*10^7$*conc[mg/ml] - 319689	0.9998	3.20	4.80
Chlorogenic acid	Phytolab, Vestenbergsgreuth, Germany	0.13–1.30	Area = $2*10^8$*conc[mg/ml] - 269699	0.9997	5.00	8.00
Salicylic acid	Merck, Darmstadt, Germany	0.16–1.60	Area = $4*10^7$*conc[mg/ml] + 44120	0.9997	1.50	2.00
Gallic acid	Phytolab, Vestenbergsgreuth, Germany	0.107-1.070	Area = $8*10^6$*conc[mg/ml]–37131	0.9999	1.90	2.80
Chrysin	Merck, Darmstadt, Germany	0.10–1.00	Area = $1*10^8$*conc[mg/ml]–82818	0.9997	3.00	5.00
Hyperoside	Phytolab, Vestenbergsgreuth, Germany	0.012–0.107	Area = $4*10^8$*conc[mg/ml]–567182	0.9986	0.60	0.90
Kaempferol	Phytolab, Vestenbergsgreuth, Germany	0.10–1.00	Area = 10^7*conc[mg/ml]–20574	0.9996	0.80	1.20
Rutoside	Phytolab, Vestenbergsgreuth, Germany	0.17–1.70	Area = $2*10^8$*conc[mg/ml]–191937	0.9996	4.00	6.00
Esculetin	Phytolab, Vestenbergsgreuth, Germany	0.06-0.62	Area = 27395*conc[mg/ml] + 394.44	0.9941	2.90	5.80
Hesperetin	Phytolab, Vestenbergsgreuth, Germany	0.10–1.00	Area = $6*10^7$*conc[mg/ml]–49247	0.9974	3.00	5.00
Luteolin	Phytolab, Vestenbergsgreuth, Germany	0.01–0.10	Area = $2*10^8$*conc[mg/ml]–2295.4	0.9977	0.05	0.07
Naringenin	Phytolab, Vestenbergsgreuth, Germany	0.16–1.60	Area = $3*10^8$*conc[mg/ml]–43443	0.9999	0.60	0.90
Quercetin	Phytolab, Vestenbergsgreuth, Germany	0.09–0.91	Area = $5*10^7$*conc[mg/ml]–9556	0.9964	0.80	1.10
Apigenin	Phytolab, Vestenbergsgreuth, Germany	0.10–0.98	Area = $2*10^8$*conc[mg/ml] + 15916	0.9999	0.20	0.30

Table S6. Retention times and main transitions for each compound identified in the hydroalcoholic extract of *O. biennis* from Macea (OHM).

OHM				
**Compound**	**Retention time, min**		**m/z and main transition**	
	**Standard**	**Separated**	**Standard**	**Separated**
Caffeic acid	13.8±1.5	14.6	177.2-180.8 > 133.7-136.4.0	179.0>135.0
Chlorogenic acid	12.0±1.5	12.8	349.5-356.5 > 189.0-192.9	353.0>191.0
Salicylic acid	23.5±1.5	23.4	135.6-138.4 > 92.0-93.9	137.0>93.0
Gallic acid	7.0±1.5	7.6	167.2-170.6 > 123.8-126.3	168.9>125.0
Chrysin	29.7±1.5	30.3	250.5-255.5 > 141.6-144.4	253.0>143.0
Esculetin	15.2±1.5	13.9	175.0-178.6 > 88.1-89.9	176.8>89.0
Hesperetin	27.1±1.5	26.7	298.0-304.0 > 162.4-165.6	301.0>164.0
Hyperoside	20.3±1.5	21.2	458.5-467.7 > 297.0-303.0	463.1>300.0
Kaempferol	27.9±1.5	27.7	282.1-287.9 > 185.1-188.9	285.0>187.0
Luteolin	26.9±1.5	25.4	284.1-289.9 > 151.5-154.5	287.0>153.0
Naringenin	26.3±1.5	27.3	268.3-273.7 > 117.8-120.1	271.0>119.0
Quercetin	25.7±1.5	26.7	297.9-303.9 > 149.5-152.5	300.9>151.0
Rutoside	20.3±1.5	21.2	602.9-615.1 > 297.0-303.0	609.0>300.0

Table S7. Retention times and main transitions for each compound identified in the hydroalcoholic extract of *O. biennis* from Vatra Dornei (OHVD).

OHVD				
**Compound**	**Retention time, min**		**m/z and main transition**	
	**Standard**	**Separated**	**Standard**	**Separated**
Caffeic acid	13.8±1.5	14.6	177.2-180.8 > 133.7-136.4.0	179.0>135.0
Chlorogenic acid	12.0±1.5	13.2	349.5-356.5 > 189.0-192.9	353.0>191.0
Salicylic acid	23.5±1.5	24.8	135.6-138.4 > 92.0-93.9	137.0>93.0
Gallic acid	7.0±1.5	7.7	167.2-170.6 > 123.8-126.3	168.9>125.0
Apigenin	28.2±1.5	28.9	266.3-271.7 > 115.8-118.1	269.0>117.0
Chrysin	29.7±1.5	30.3	250.5-255.5 > 141.6-144.4	253.0>143.0
Esculetin	15.2±1.5	14.0	175.0-178.6 > 88.1-89.9	176.8>89.0
Hesperetin	27.1±1.5	26.8	298.0-304.0 > 162.4-165.6	301.0>164.0
Hyperoside	20.3±1.5	21.2	458.5-467.7 > 297.0-303.0	463.1>300.0
Kaempferol	27.9±1.5	27.9	282.1-287.9 > 185.1-188.9	285.0>187.0
Luteolin	26.9±1.5	25.6	284.1-289.9 > 151.5-154.5	287.0>153.0
Naringenin	26.3±1.5	27.4	268.3-273.7 > 117.8-120.1	271.0>119.0
Quercetin	25.7±1.5	26.9	297.9-303.9 > 149.5-152.5	300.9>151.0
Rutoside	20.3±1.5	21.2	602.9-615.1 > 297.0-303.0	609.0>300.0

Table S8. Retention times and main transitions for each compound identified in the aqueous extract of *O. biennis* from Macea (OAM).

OAM				
**Compound**	**Retention time, min**		**m/z and main transition**	
	**Standard**	**Separated**	**Standard**	**Separated**
Caffeic acid	13.8±1.5	14.5	177.2-180.8 > 133.7-136.4.0	179.0>135.0
Chlorogenic acid	12.0±1.5	12.9	349.5-356.5 > 189.0-192.9	353.0>191.0
Salicylic acid	23.5±1.5	24.6	135.6-138.4 > 92.0-93.9	137.0>93.0
Gallic acid	7.0±1.5	7.7	167.2-170.6 > 123.8-126.3	168.9>125.0
Esculetin	15.2±1.5	14.1	175.0-178.6 > 88.1-89.9	176.8>89.0
Hesperetin	27.1±1.5	26.7	298.0-304.0 > 162.4-165.6	301.0>164.0
Hyperoside	20.3±1.5	21.2	458.5-467.7 > 297.0-303.0	463.1>300.0
Naringenin	26.3±1.5	27.4	268.3-273.7 > 117.8-120.1	271.0>119.0
Quercetin	25.7±1.5	26.7	297.9-303.9 > 149.5-152.5	300.9>151.0
Rutoside	20.3±1.5	21.2	602.9-615.1 > 297.0-303.0	609.0>300.0

Table S9. Retention times and main transitions for each compound identified in the aqueous extract of *O. biennis* from Vatra Dornei (OAVD).

OAVD				
**Compound**	**Retention time, min**		**m/z and main transition**	
	**Standard**	**Separated**	**Standard**	**Separated**
Chlorogenic acid	12.0±1.5	12.9	349.5-356.5 > 189.0-192.9	353.0>191.0
Salicylic acid	23.5±1.5	24.7	135.6-138.4 > 92.0-93.9	137.0>93.0
Gallic acid	7.0±1.5	7.7	167.2-170.6 > 123.8-126.3	168.9>125.0
Chrysin	29.7±1.5	30.5	250.5-255.5 > 141.6-144.4	253.0>143.0
Hyperoside	20.3±1.5	21.2	458.5-467.7 > 297.0-303.0	463.1>300.0

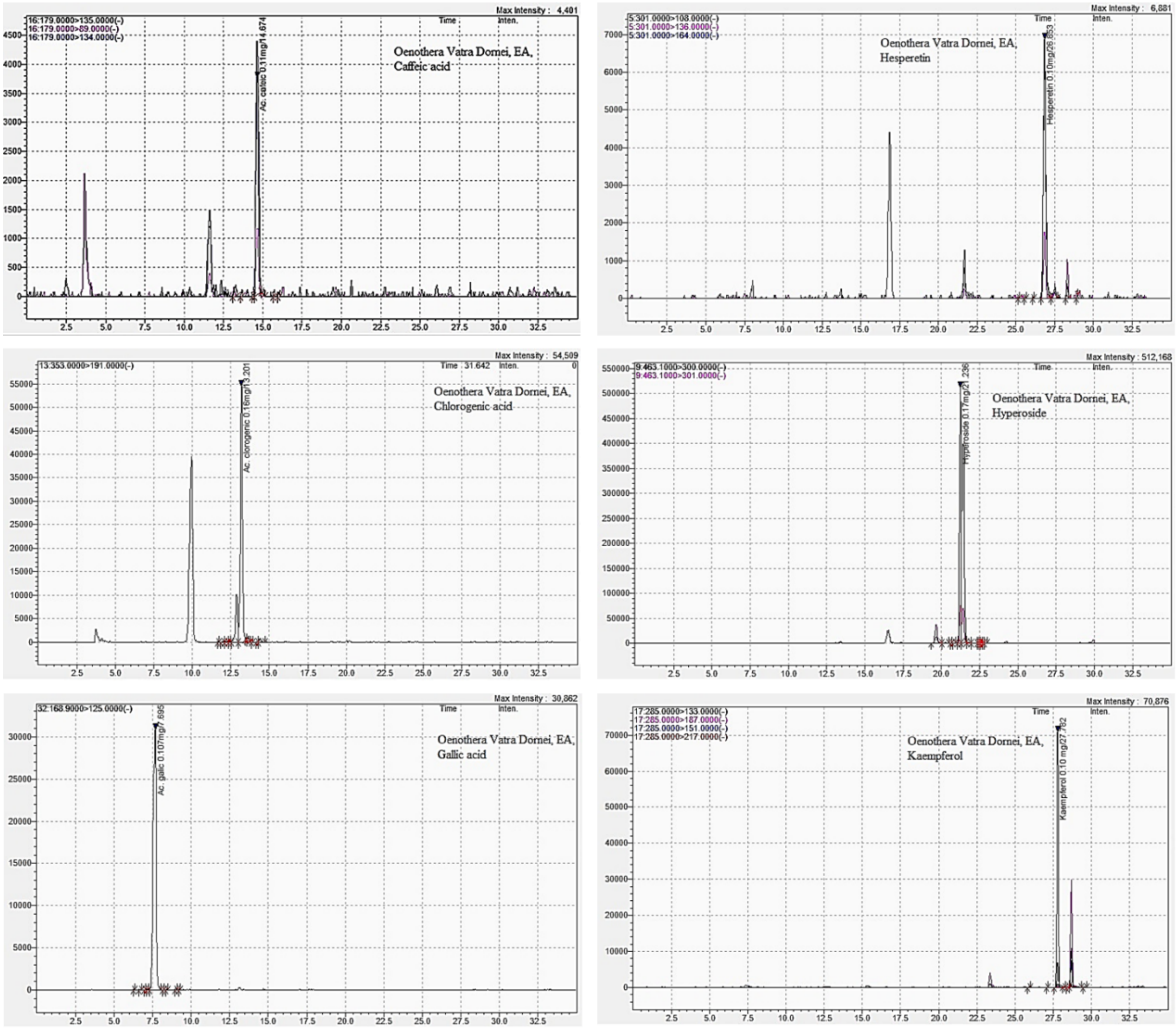

Figure S4. LC/MS chromatogram of the hydroalcoholic extract obtained from *O. biennis* L. plants from Vatra Dornei (OHVD) showing (left to right, top to bottom): caffeic acid, hesperetin, chlorogenic acid, hyperoside, gallic acid, and kaempferol.

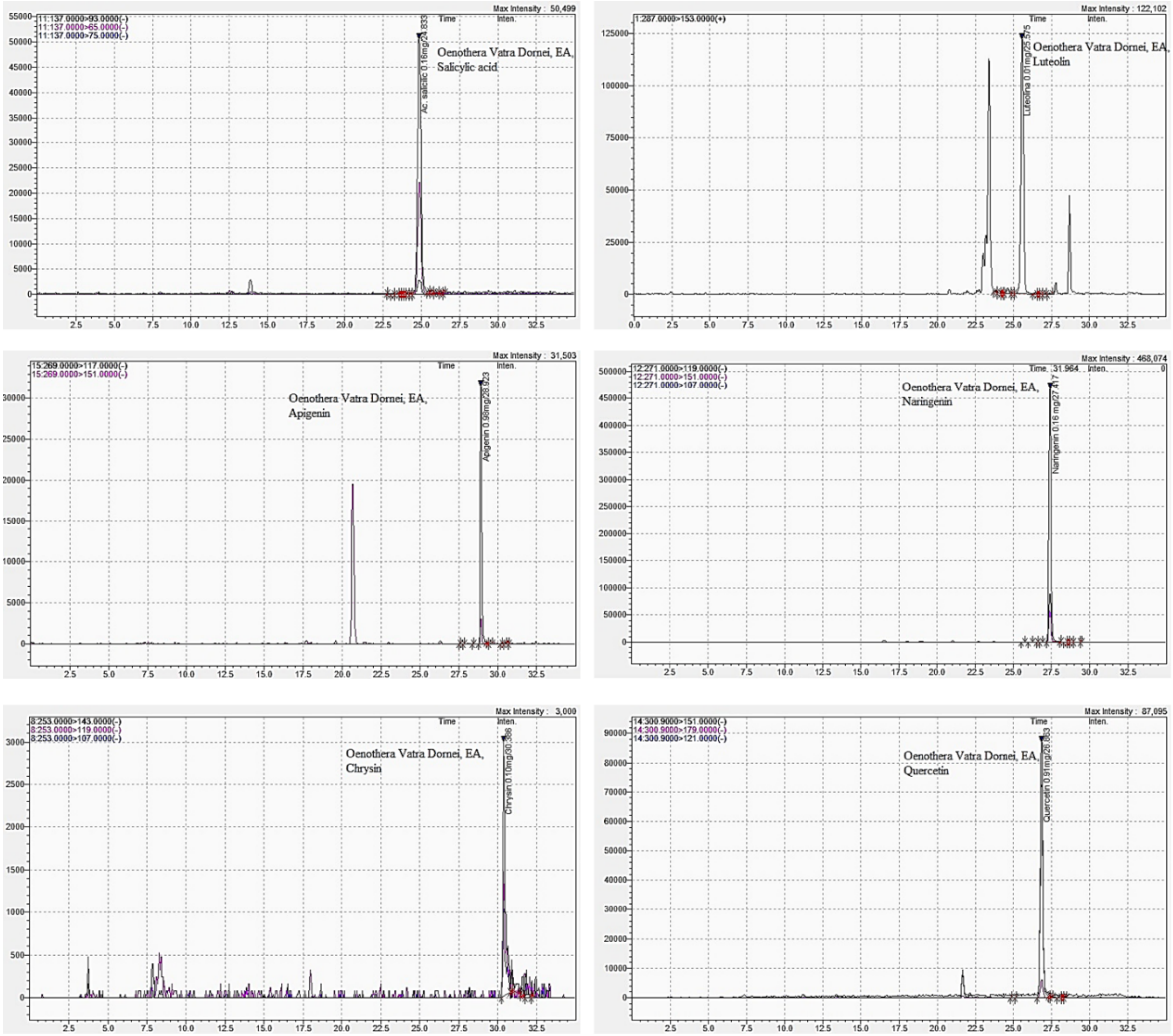

Figure S5. LC/MS chromatogram of the hydroalcoholic extract obtained from *O. biennis* L. plants from Vatra Dornei (OHVD) showing (left to right, top to bottom): salicylic acid, luteolin, apigenin, naringenin, chrysin, and quercetin.

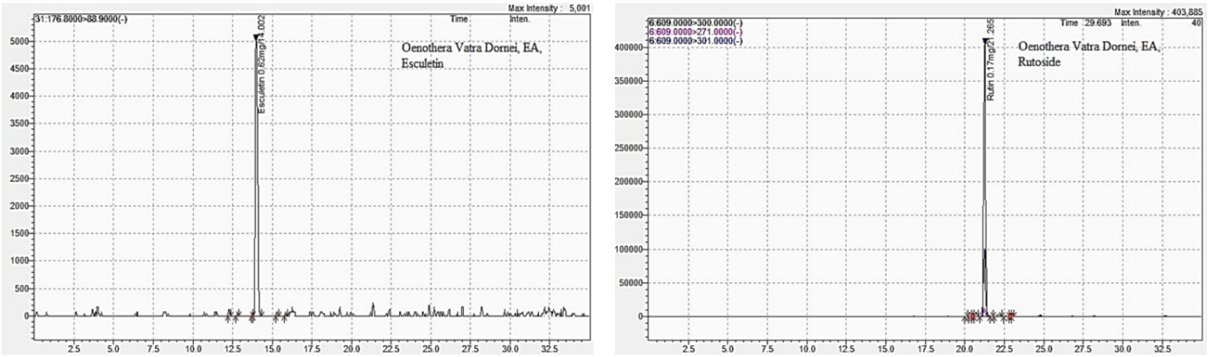

Figure S6. LC/MS chromatogram of the hydroalcoholic extract obtained from *O. biennis* L. plants from Vatra Dornei (OHVD) showing (left to right): esculetin and rutoside.

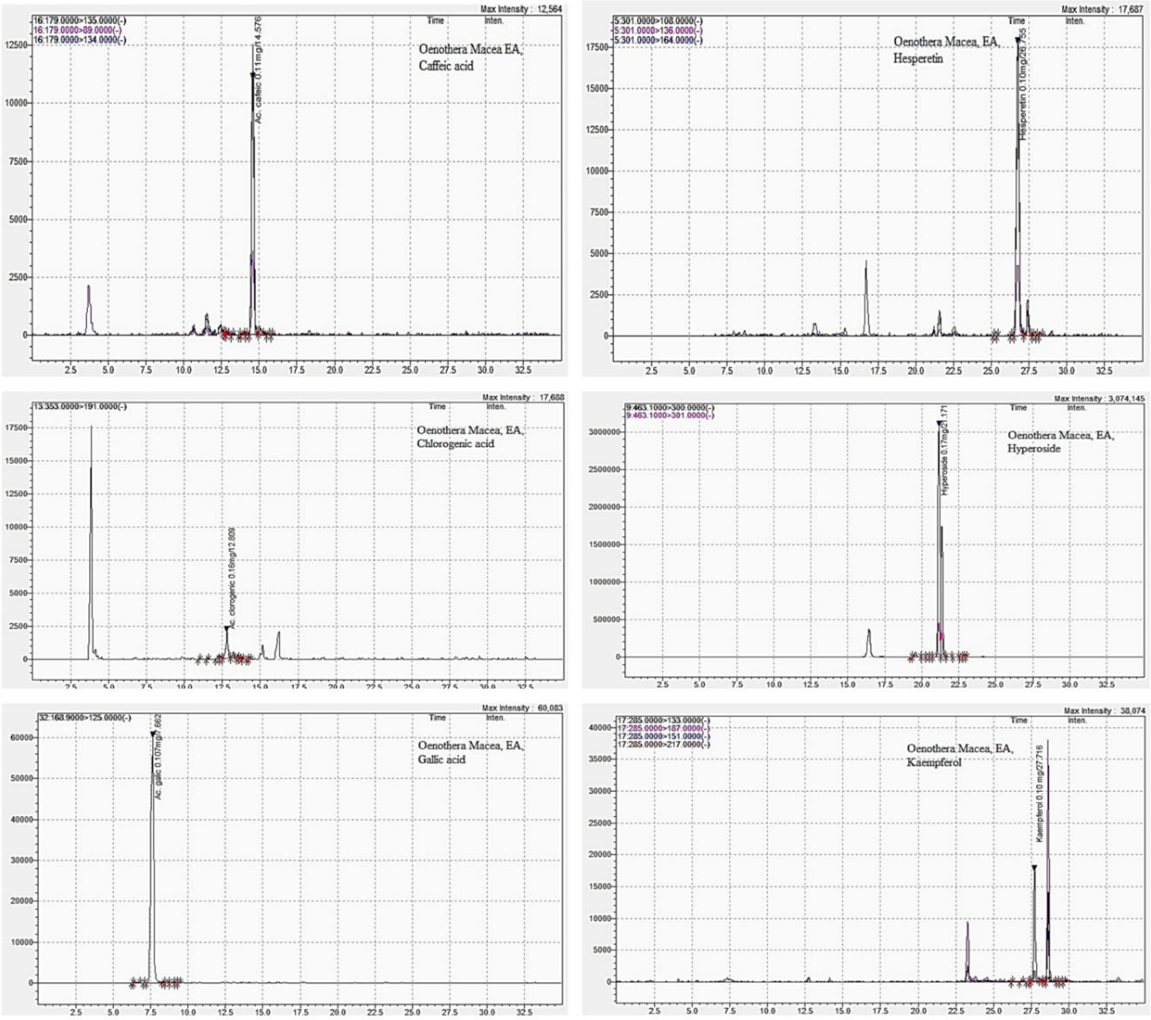

Figure S7. LC/MS chromatogram of the hydroalcoholic extract obtained from *O. biennis* L. plants from Macea (OHM) showing (left to right, top to bottom): caffeic acid, hesperetin, chlorogenic acid, hyperoside, gallic acid, and kaempferol.

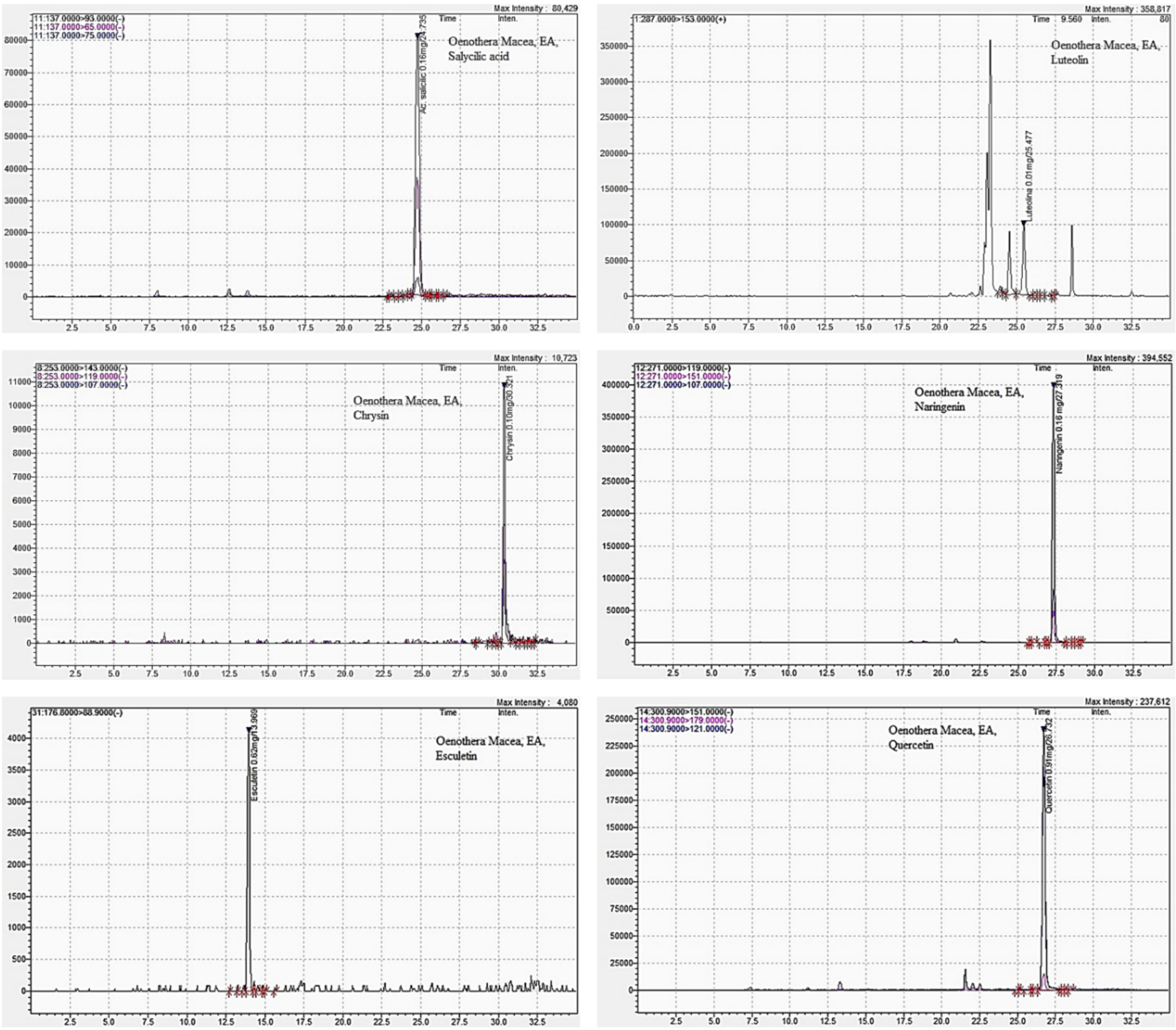

Figure S8. LC/MS chromatogram of the hydroalcoholic extract obtained from *O. biennis* L. plants from Macea (OHM) showing (left to right, top to bottom): salycilic acid, luteolin, chrysin, naringenin, esculetin, and quercetin.

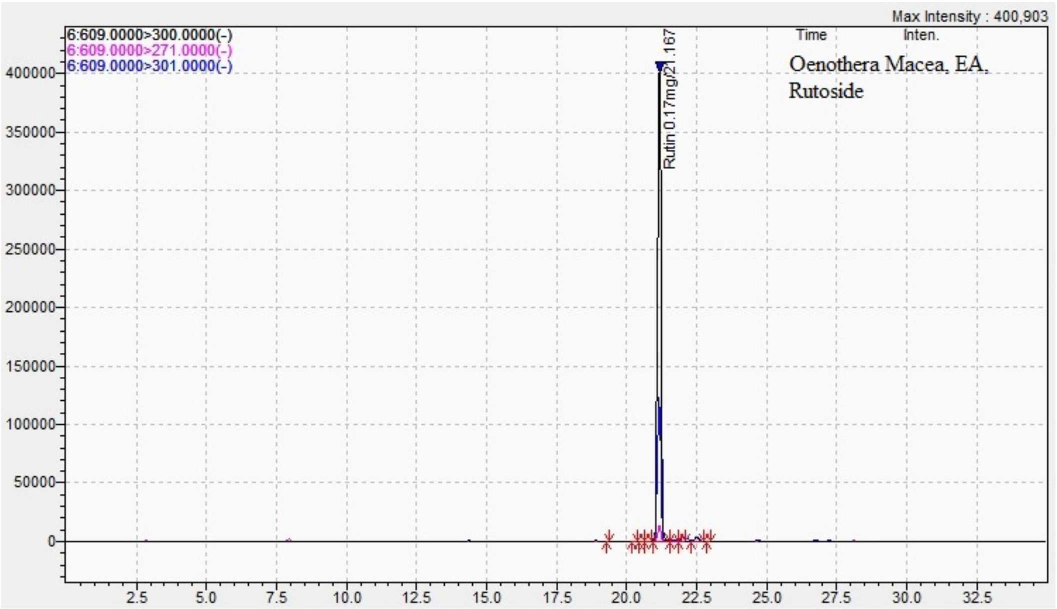

Figure S9. LC/MS chromatogram of the hydroalcoholic extract obtained from *O. biennis* L. plants from Macea (OHM) showing rutoside.

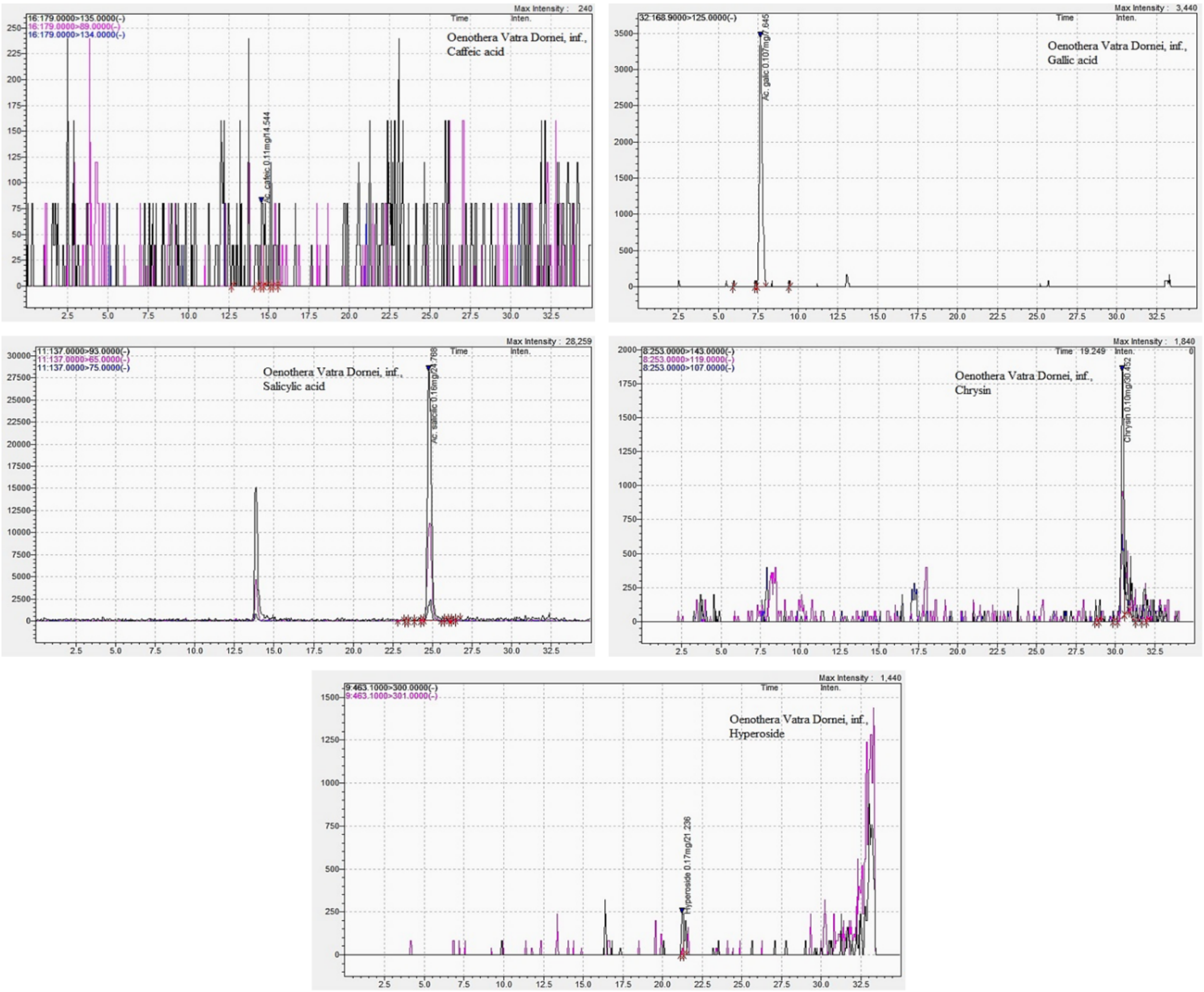

Figure S10. LC/MS chromatogram of the aqueous extract obtained from *O. biennis* L. plants from Vatra Dornei (OAVD) showing (left to right, top to bottom): caffeic acid, gallic acid, salicylic acid, chrysin, and hyperoside.

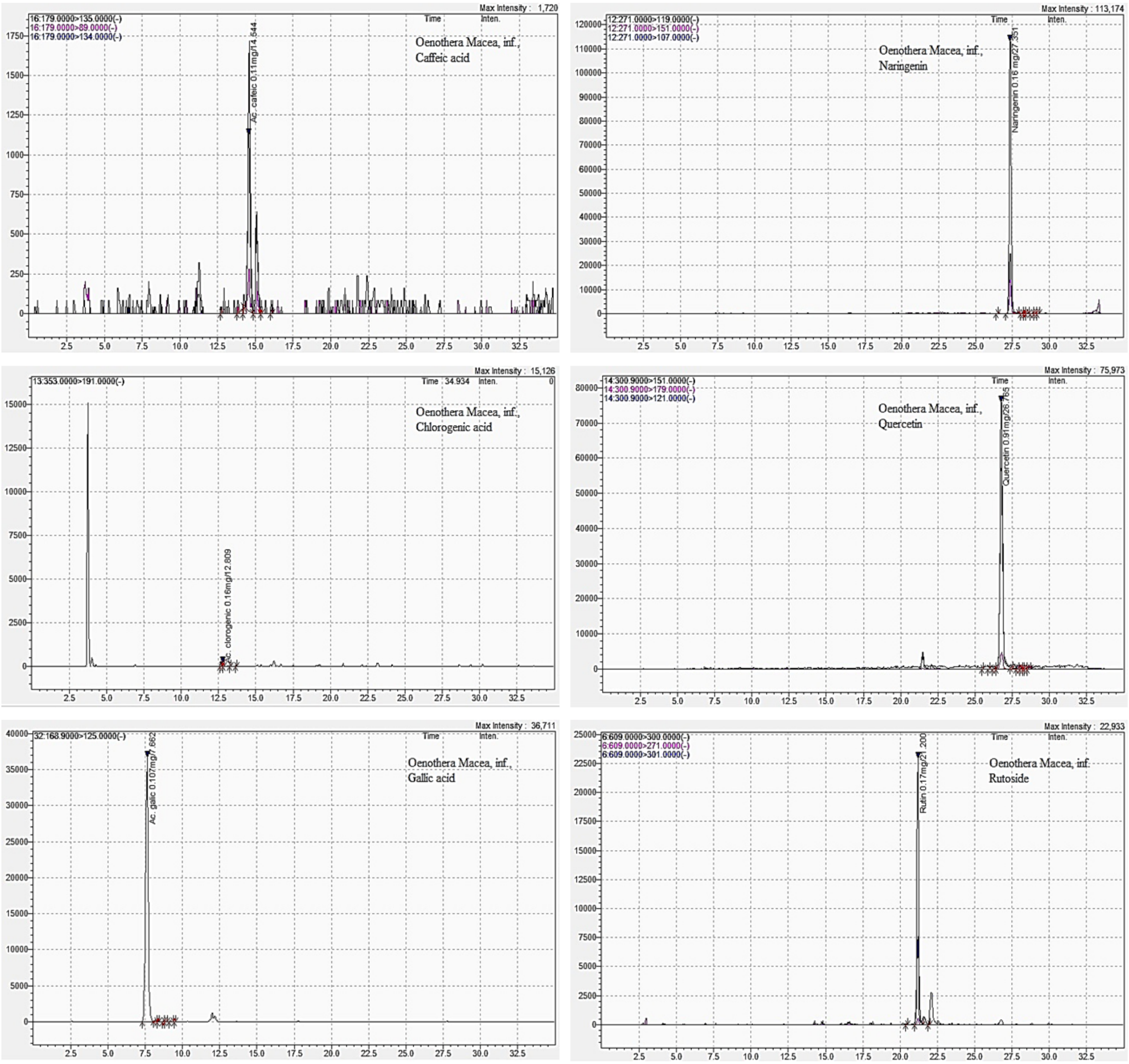

Figure S11. LC/MS chromatogram of the aqueous extract obtained from *O. biennis* L. plants from Macea (OAM) showing (left to right, top to bottom): caffeic acid, naringenin, chlorogenic acid, quercetin, gallic acid, and rutoside.

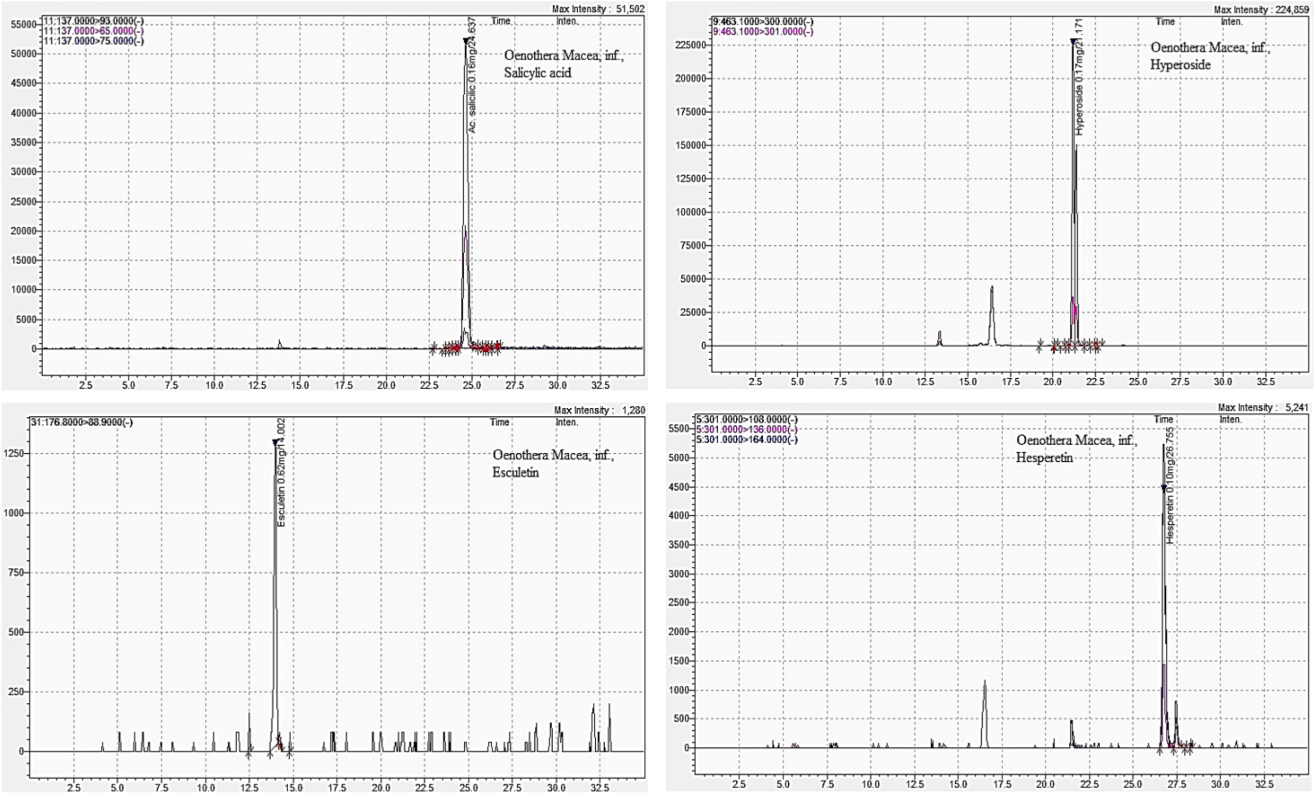

Figure S12. LC/MS chromatogram of the aqueous extract obtained from *O. biennis* L. plants from Macea (OAM) showing (left to right, top to bottom): salicylic acid, hyperoside, esculetin, and hesperetin.

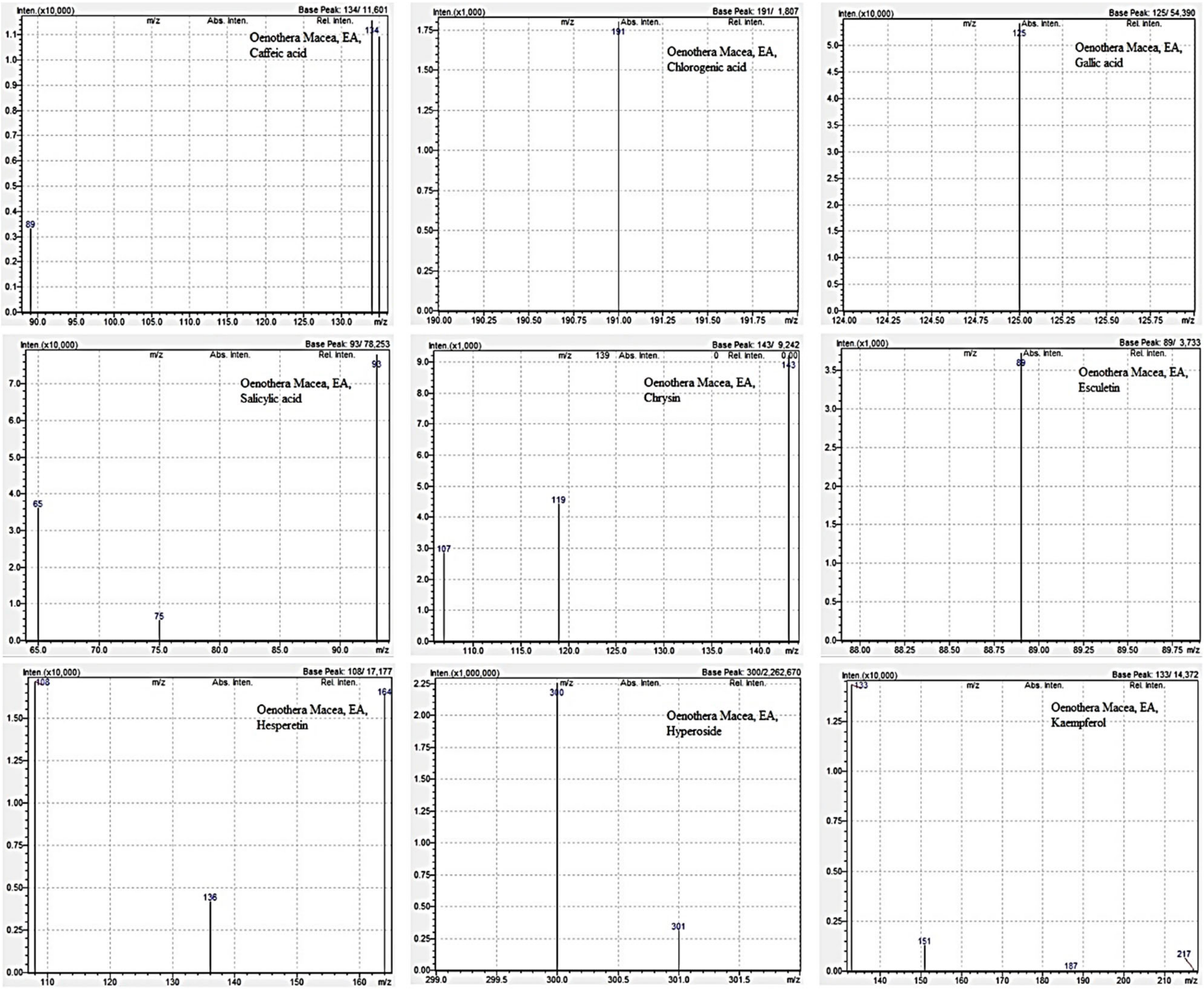

Figure S13. MS spectra of separated compounds from the hydroalcoholic extract of *O. biennis* L. plants from Macea (OHM) showing (left to right, top to bottom): caffeic acid, chlorogenic acid, gallic acid, salicylic acid, chrysin, esculetin, hesperetin, hyperoside, and kaempferol.

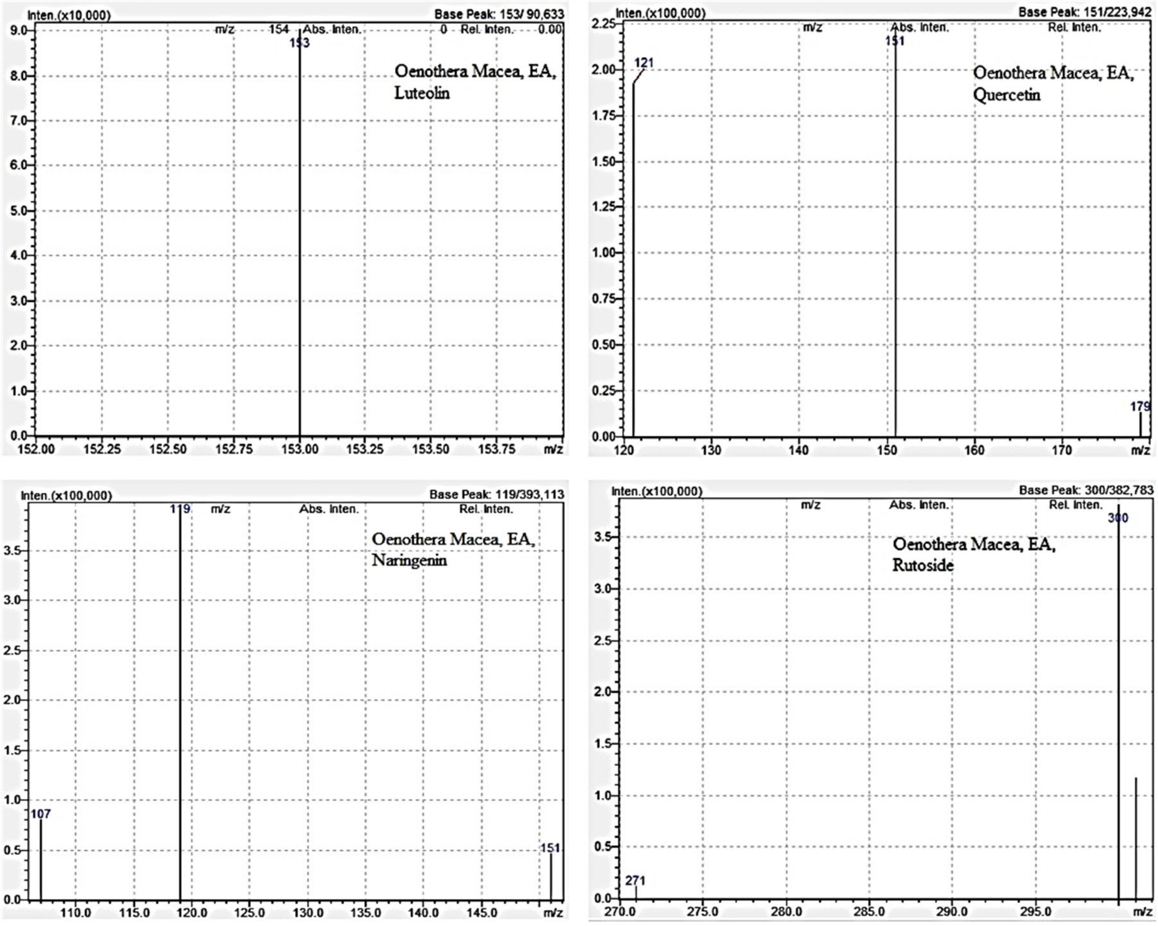

Figure S14. MS spectra of separated compounds from the hydroalcoholic extract of *O. biennis* L. plants from Macea (OHM) showing (left to right, top to bottom): luteolin, quercetin, naringenin, and rutoside.

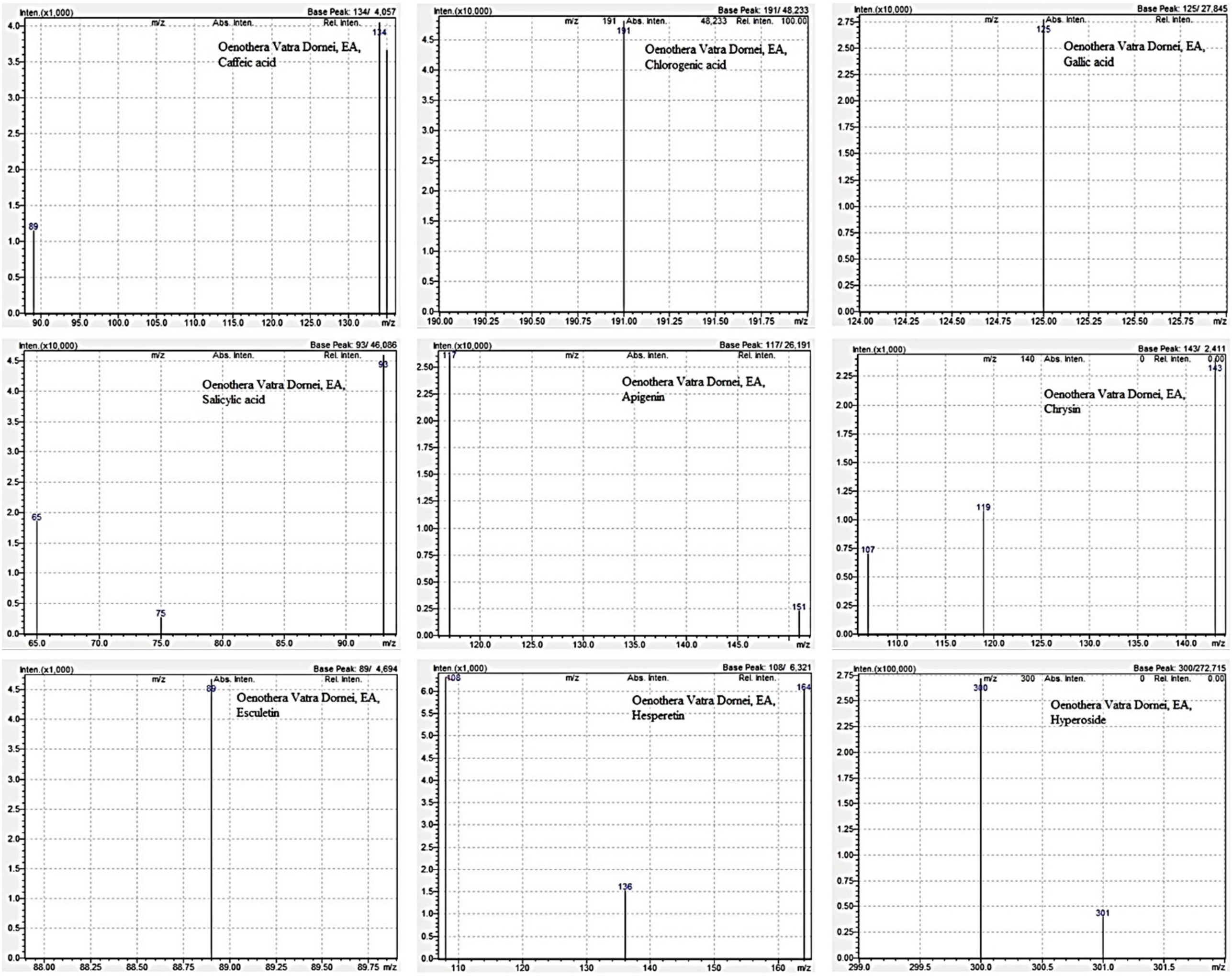

Figure S15. MS spectra of separated compounds from the hydroalcoholic extract of *O. biennis* L. plants from Vatra Dornei (OHVD) showing (left to right, top to bottom): caffeic acid, chlorogenic acid, gallic acid, salicylic acid, apigenin, chrysin, esculetin, hesperetin, and hyperoside.

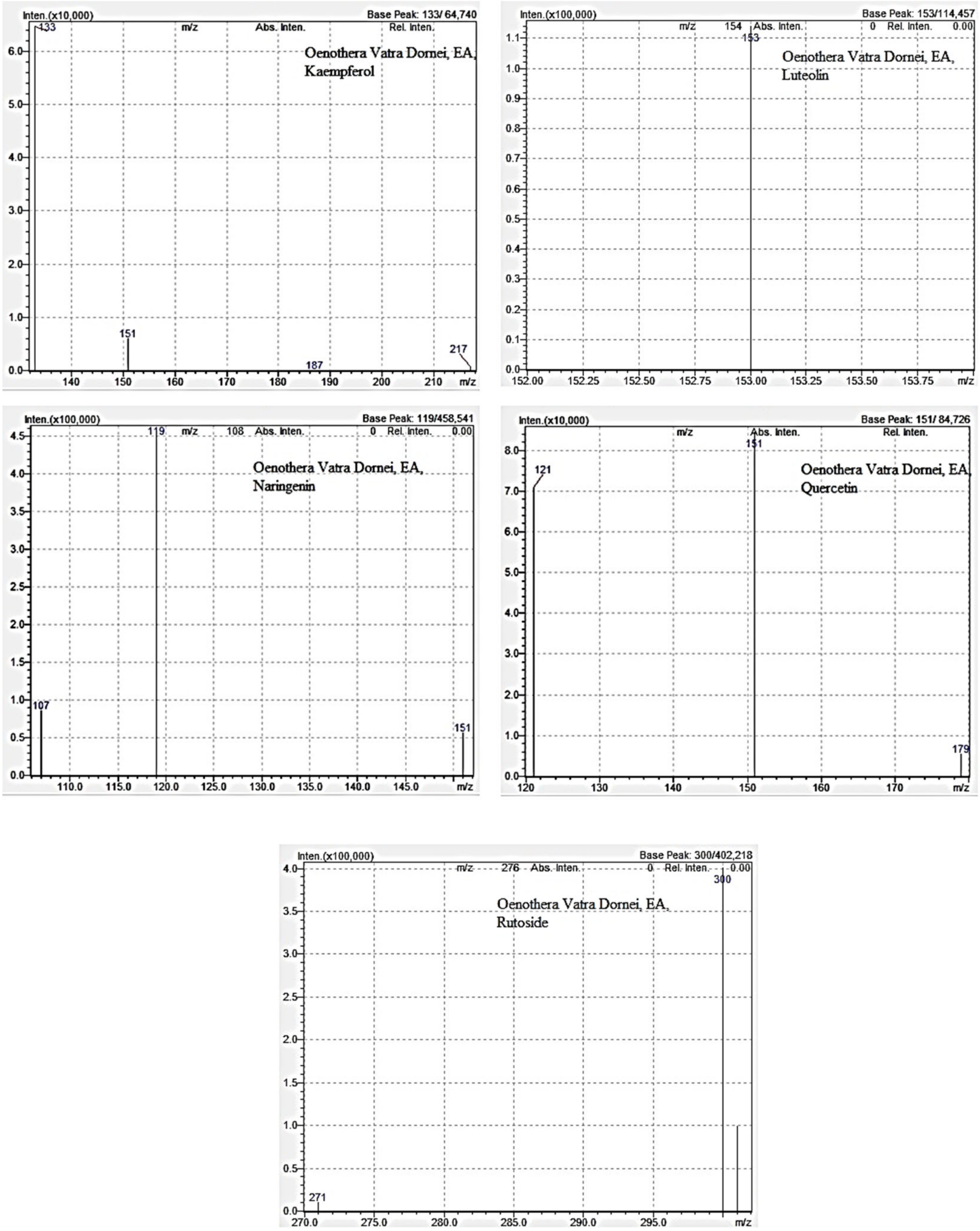

Figure S16. MS spectra of separated compounds from the hydroalcoholic extract of *O. biennis* L. plants from Vatra Dornei (OHVD) showing (left to right, top to bottom): kaempferol, luteolin, naringenin, quercetin, and rutoside.

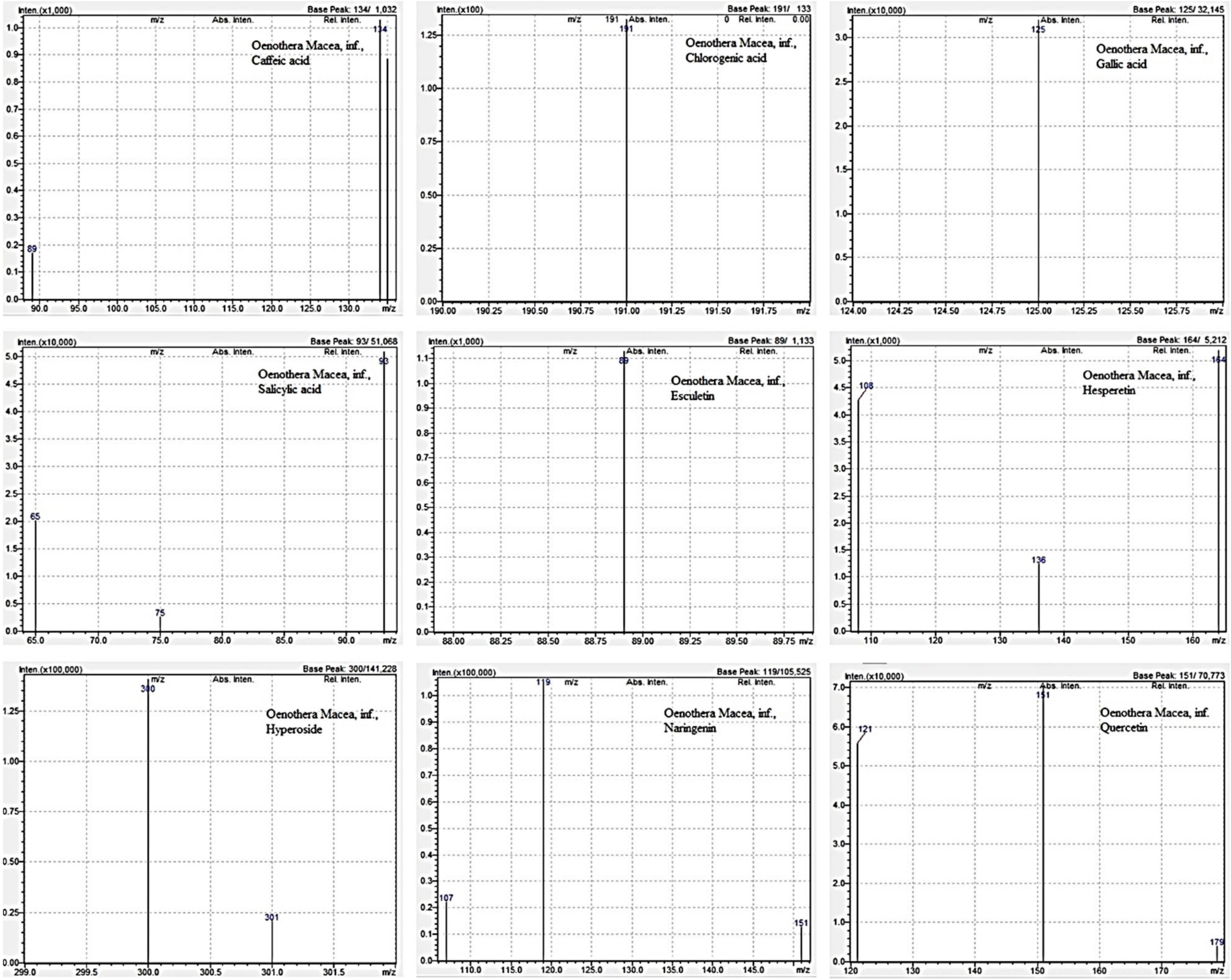

Figure S17. MS spectra of separated compounds from the aqueous extract of *O. biennis* L. plants from Macea (OAM) showing (left to right, top to bottom): caffeic acid, chlorogenic acid, gallic acid, salicylic acid, esculetin, hesperetin, hyperoside, naringenin, and quercetin.

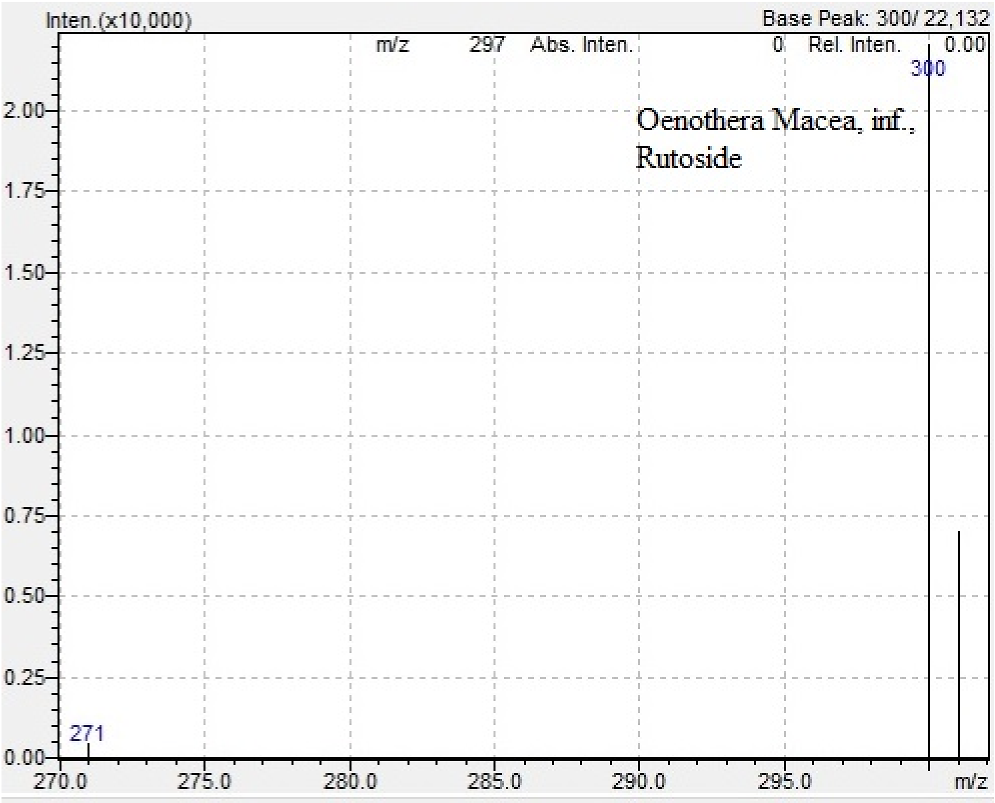

Figure S18. MS spectra of separated rutoside from the aqueous extract of *O. biennis* L. plants from Macea (OAM).

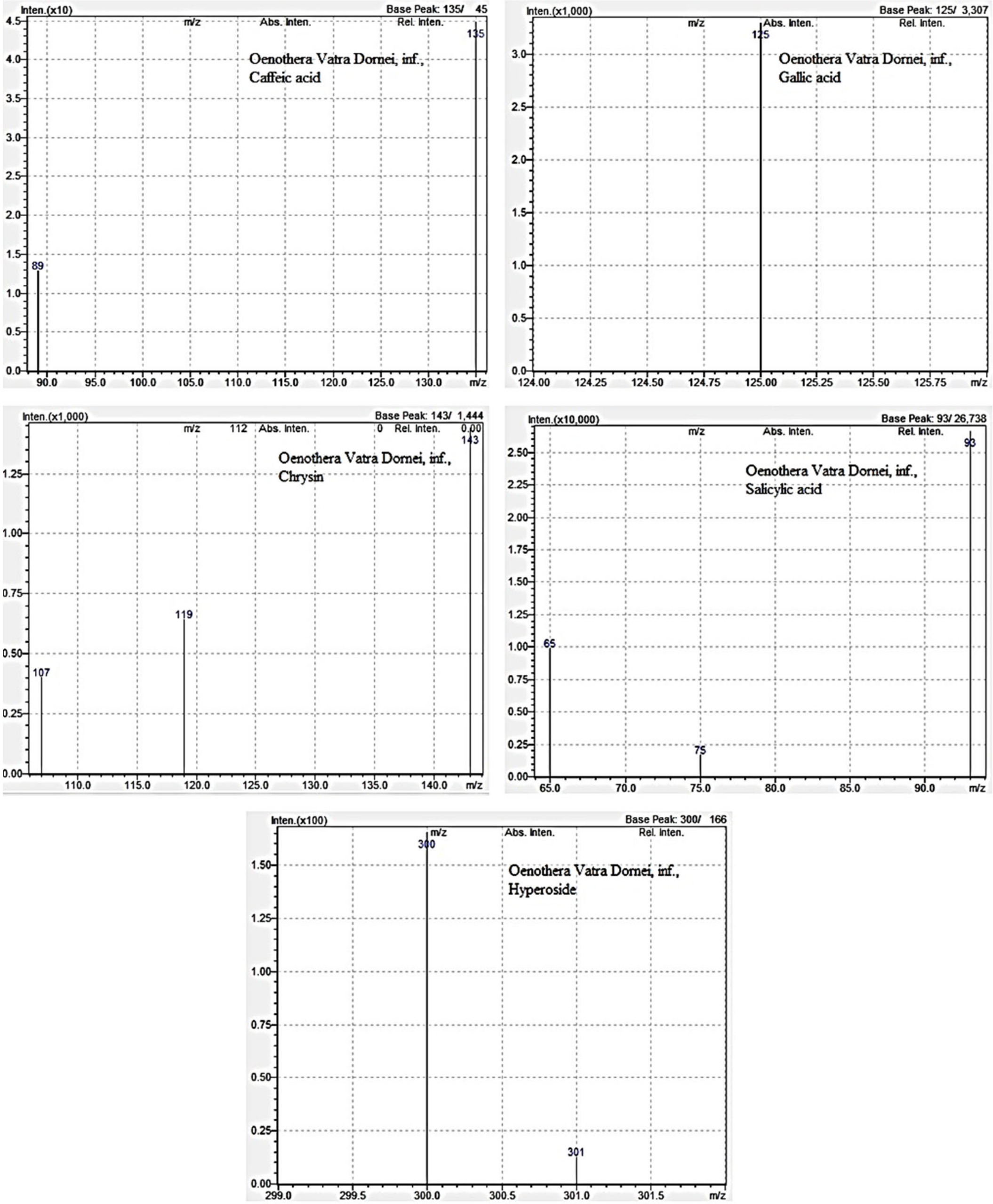

Figure S19. MS spectra of separated compounds from the aqueous extract of *O. biennis* L. plants from Vatra Dornei (OAVD) showing (left to right, top to bottom): caffeic acid, gallic acid, chrysin, salicylic acid, and hyperoside.
